# Ultrasound-guided single thoracic paravertebral nerve block and erector spinae plane block for perioperative analgesia in thoracoscopic pulmonary lobectomy: a randomized controlled trial

**DOI:** 10.1186/s13244-021-01151-x

**Published:** 2022-01-28

**Authors:** Jian-wen Zhang, Xiao-yue Feng, Jing Yang, Zhi-hao Wang, Zhe Wang, Li-ping Bai

**Affiliations:** 1 Department of Anesthesiology, Shanxi Bethune Hospital, Shanxi Academy of Medical Sciences, Tongji Shanxi Hospital, Third Hospital of Shanxi Medical University, No. 99 of Longcheng Street, Xiaodian District, Taiyuan, 030032 China; 2 Department of Anesthesiology, Tongji Hospital, Tongji Medical College, Huazhong University of Science and Technology, Wuhan, 430030 China; 3 Department of Anesthesiology, Third Hospital of Shanxi Medical University, Shanxi Bethune Hospital, Shanxi Academy of Medical University, Tongji Shanxi Hospital, Taiyuan, 030032 China

**Keywords:** Thoracic paravertebral nerve block, Erector spinae plane block, Thoracoscopic surgery, Pulmonary lobectomy, Analgesia

## Abstract

**Objective:**

To explore the effect of a single preoperative ultrasound-guided thoracic paravertebral nerve block (TPVB) and erector spinae plane block (ESPB) for perioperative analgesia in thoracoscopic pulmonary lobectomy.

**Methods:**

Seventy-two patients aged 40–70 years who underwent thoracoscopic pulmonary lobectomy under general anesthesia were enrolled and randomly divided into the control group (Group C), the TPVB group (Group T) and the ESPB group (Group E). The primary observation indicators included the visual analogue scale (VAS) at 1, 6, 12, 24, and 48 h postoperatively at rest and with a cough. The secondary observation indicators included the intraoperative sufentanil consumption, anesthesia awakening time and extubation time, the sufentanil consumption in the analgesic pump, and flurbiprofen ester consumption for remedial analgesia within 48 h after surgery and the incidence of postoperative adverse events.

**Results:**

The intraoperative sufentanil consumption, anesthesia awakening time, and extubation time were lower in groups T and E than those in group C (*p* < 0.05). Patients in group T had lower VAS scores at rest and with a cough at 1, 6, and 12 h postoperatively than in group C at the same time points (*p* < 0.05). The VAS scores at rest at 1 and 6 h postoperatively and coughing status at 1, 6, and 12 h postoperatively were lower in group E than in group C at the same time points (*p* < 0.05).

**Conclusion:**

The ultrasound-guided preoperative single TPVB and ESPB for thoracoscopic pulmonary lobectomy could both reduce the postoperative pain VAS score and reduce the dose of perioperative sufentanil and postoperative remedial analgesics.

## Key points

- Preoperative ultrasound-guided single TPVB and ESPB for thoracoscopic pulmonary lobectomy could reduce the VAS of postoperative pain in patients and decrease the perioperative sufentanil and postoperative remedial analgesic medications.

## Introduction

Thoracic paravertebral nerve block (TPVB) is a nerve block technique in which the local anesthetic is injected into the thoracic paravertebral space to block the thoracic spinal nerve, its branches, and the sympathetic trunk, providing analgesia comparable to that of a thoracic epidural block. Sen et al. [1] found that preoperative TPVB significantly reduced the dose of perioperative analgesics, decreased the postoperative serum concentrations of vascular endothelial growth factor (VEGF) and transforming growth factor-β (TGF-β), and reduced the risk of invasion, proliferation, and metastasis of the residual lung cancer cells in patients undergoing thoracoscopic surgery. Kang et al. [2] found that in patients with lung cancer undergoing thoracoscopic radical surgery, the preoperative TPVB at T4–5 and T6–7 resulted in significantly lowered pain scores and oxycodone doses within 24 h after surgery, and patients had significantly better sleep quality and higher completion rates of walking tests postoperatively.

Erector spinae plane block (ESPB) is a new regional anesthesia technique recently described by Forero et al. [3] to treat chronic thoracic neuropathic pain. ESPB is performed by depositing local anesthetic in the fascial plane, deeper than the erector spinae muscle at the tip of the transverse process. ESPB can provide effective postoperative analgesia for breast surgery [4–6] and thoracic surgery [7–10] when performed at the thoracic vertebra 4–5 level, and thoracic vertebra 7–8 level for abdominal surgery [11–14], and the fourth lumbar vertebra level for lower limb surgery [11]. When ESPB is performed bilaterally, it has been reported to be as effective as thoracic epidural analgesia [7].

A randomized controlled prospective study was designed to investigate the effectiveness of ultrasound-guided preoperative single TPVB and ESPB for perioperative analgesia in thoracoscopic pulmonary lobectomy. It was hypothesized that a single preoperative ultrasound-guided TPVB and ESPB for thoracoscopic pulmonary lobectomy could reduce the visual analogue score (VAS) of postoperative pain and reduce the dose of perioperative sufentanil and postoperative remedy analgesics.

## Materials and methods

### General materials

The present study was reviewed and approved by the Ethics Committee of Shanxi Bethune Hospital (approval number: YXLL-2020-071) and registered with the China Clinical Trials Registry (registration number: ChiCTR2100043516). All patients participating in the present study signed informed consent. Seventy-two patients who underwent elective thoracoscopic pulmonary lobectomy under general anesthesia between November 2020 and February 2021 at Shanxi Bethune Hospital were enrolled.

The inclusion criteria were: patients aged 40–70 years, with a body mass index (BMI) of 18–30 kg/m^2^, and patients with the American Society of Anesthesiologists (ASA) classification I–II. The exclusion criteria were: patients with spinal deformities, infection at or near the puncture site, abnormal coagulation, a history of allergy to local anesthetics, a history of psychiatric disorders, inability to cooperate, or refusal to sign an informed consent. The present study was a prospective randomized controlled study, and the SPSS 9.4 statistical software was applied to generate a random number table. The enrolled subjects were randomly divided into the control group (group C), the TPVB group (group T), and the ESPB group (group E), with 24 cases in each group. The entire study procedure and operations were in accordance with the Declaration of Helsinki. The attending anesthesiologists in the present study were made aware of the patient groupings, and the patients and data collection recorders were blind to the groups. All patients in the present study were operated on by the same thoracic team.

### Anesthesia

All subjects were routinely fasted for 8 h with water deprivation for 4 h before surgery, and no pre-anesthetic medication was administered. After entering the operating room, the patient was routinely connected to an M1205A monitor to monitor the electrocardiogram (ECG) continuously, non-invasive blood pressure (NIBP), heart rate (HR), pulse oxygen saturation (SpO2), and breathing rate (BR). The EEG Bispectral index (BIS) was monitored by connecting to the EEG Bispectral index detector.

After the patient entered the operating room, the peripheral vein of the upper extremity was punctured for intravenous infusion of Lactated Ringer’s solution, and oxygen was administered by face mask with an oxygen flow rate of 5 L/min. All patients were induced by the same method of routine intravenous rapid anesthesia: 0.05 mg/kg of midazolam, 0.5 μg/kg of sufentanil, 0.6 mg/kg of rocuronium, and 0.3 mg/kg of etomidate were given sequentially by intravenous infusion. Oxygen was administered under face-mask pressurized ventilation for 3 min, and a double-lumen tracheal tube was intubated via a visual laryngoscope. After the fiberoptic bronchoscope assisted in positioning to make sure that the tracheal tube was correctly positioned, the anesthetic ventilator was connected to perform mechanical ventilation with a tidal volume of 6–8 ml/kg and a BR of 12/min. Before the start of surgery, lung isolation was performed with unilateral lung ventilation, and the ventilation frequency and tidal volume were adjusted to maintain an end-tidal carbon dioxide partial pressure (PetCO2) of 35–45 mmHg.

**Fig. 1:**
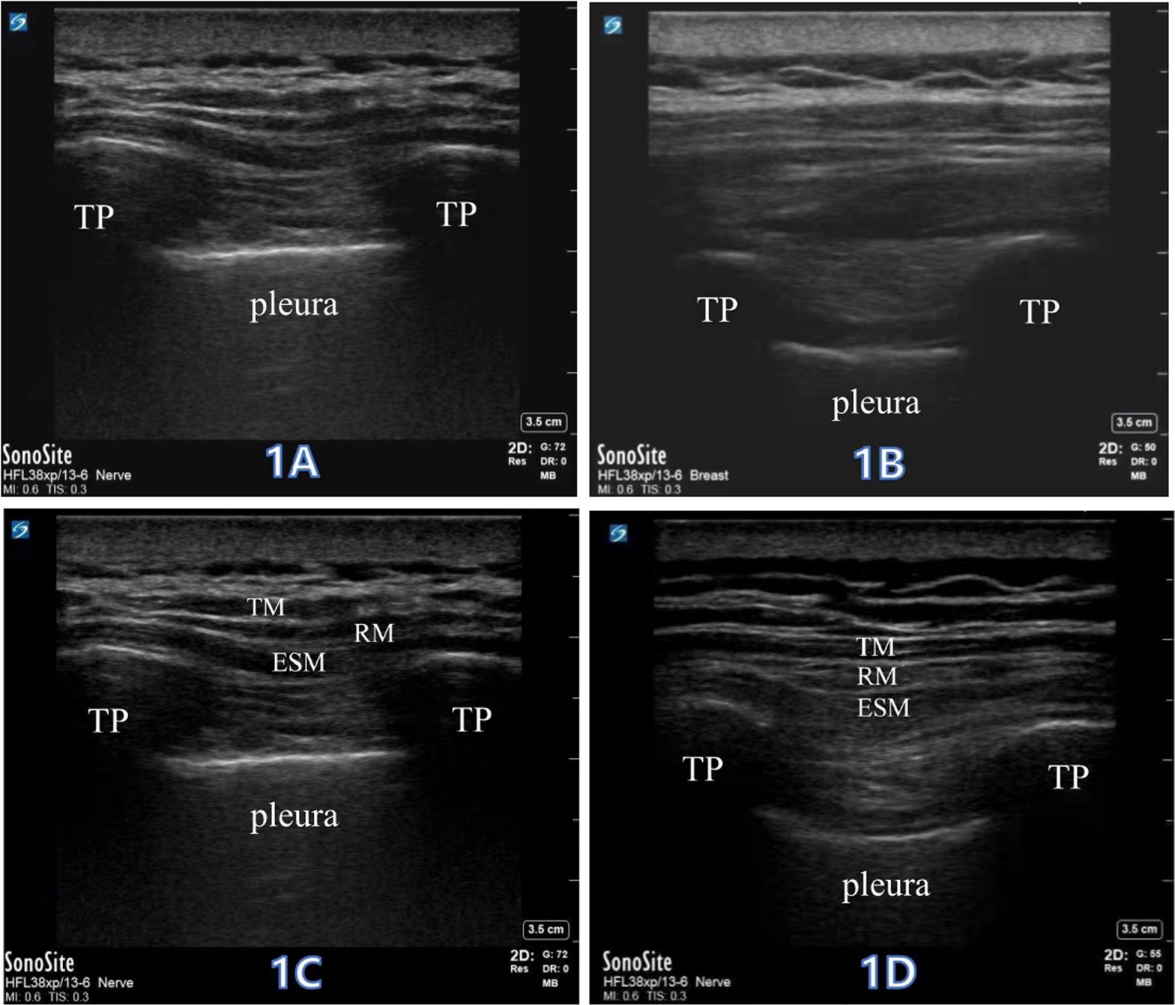
The ultrasound-guided thoracic paravertebral nerve block and erector spinae plane block. **a**, **b** An ultrasound high-frequency line array probe was used to scan at approximately 2–2.5 cm next to T4–5 spinous process in the median sagittal position. The transverse process (TP), pleura, and thoracic paravertebral space could be clearly visualized under the ultrasound, and 30 ml of 0.5% ropivacaine hydrochloride was injected into T4–5 paravertebral spaces using an out-of-plane approach technique. **a** Ultrasound-guided thoracic paravertebral nerve block before injection. **b** After ultrasound-guided thoracic paravertebral nerve block by local anesthetic injection, the pleura would be pushed downward. **c**, **d** An ultrasound high-frequency line array probe was used to scan at approximately 2–2.5 cm next to T4–5 spinous process in the median sagittal position. With an out-of-plane approach technique, trapezius muscle (TM), rhomboid muscle (RM) and erector spinae muscle (ESM) were sequentially breached, and 30 ml of 0.5% ropivacaine hydrochloride was injected into the deep surface between erector spinae muscle and T4 and T5 transverse processes (TP). **c** Ultrasound-guided erector spinae plane block before injection. **d** Ultrasound-guided erector spinae plane block after injection

Group T: after induction of anesthesia, the patient was placed in the lateral position with the affected side in the superior position, and an ultrasound high-frequency line array probe (Sonosite S-nerve, USA) was used to scan at approximately 2–2.5 cm next to T4–5 spinous process in the median sagittal position. The transverse process, pleura, and thoracic paravertebral space could be clearly visualized under the ultrasound, and 30 ml of 0.5% ropivacaine hydrochloride (AstraZeneca, lot number ps05072) was injected into T4–5 paravertebral spaces using an out-of-plane approach technique (Fig. 1).

Group E: after induction of anesthesia, the patient was placed in the lateral position with the affected side in the superior position, and an ultrasound high-frequency line array probe (Sonosite S-nerve, USA) was used to scan at approximately 2–2.5 cm next to T4–5 spinous process in the median sagittal position. With an out-of-plane approach technique, the trapezius, rhomboid, and erector spinae were sequentially breached, and 30 ml of 0.5% ropivacaine hydrochloride (AstraZeneca, lot no. ps05072) was injected into the deep surface between erector spinae and T4 and T5 transverse processes (Fig. 1).

### The nerve block was not conducted in Group C

Perioperative anesthesia was maintained by total intravenous anesthesia. Propofol infusion was adjusted to maintain BIS between 40 and 60. The sufentanil infusion was adjusted to maintain the mean arterial pressure (MAP) between ± 20% of the preoperative baseline level. Additional rocuronium was given on demand. During the perioperative period, when the systolic blood pressure (SBP) was < 80% of the baseline level or SBP < 90 mmHg, 6 mg of ephedrine was administered intravenously. When the HR was < 80% of the baseline level or HR < 60 beats/min, 0.5 mg of atropine was administered intravenously.

The administration of propofol and sufentanil was discontinued at the time of suturing in all patients, and an intravenous injection of 1 mg of butorphanol and 12.5 mg of dolasetron mesylate were given. After consciousness and spontaneous respiration were fully restored in the patient, the tracheal tube was extracted, and the patient was escorted to the post-anesthesia recovery room for observation. With the operation’s completion, a transvenous patient-controlled analgesia pump was turned on. The formulation of the pump was: 100 μg of sufentanil and 25 mg of dolasetron mesylate were diluted to 100 ml with 0.9% normal saline. The settings of the analgesic pump were: a loading dose of 2 ml, a background dose of 1 ml/h, a compression dose of 2 ml, and a lock time of 15 min. If the VAS was ≥ 4 at rest or with a cough, 50 mg of flurbiprofen ester was administered for remedy analgesia.

### Observation indicators

#### The primary observation indicators

The VAS was observed at rest and with a cough at 1, 6, 12, 24, and 48 h postoperatively (0 for no pain; 10 points for intolerable severe pain; < 4 points for mild pain; 4–7 points for moderate pain; > 7 points for severe pain).

#### The secondary observation indicators

- The intraoperative sufentanil consumption, anesthesia awakening time (the time from the termination of the anesthetics to the time when the patient could open their eyes by calling), and extubation time (the time from the termination of the anesthetics to the time when the tracheal tube was extracted).
- The sufentanil consumption in the analgesic pump and the dose of flurbiprofen ester for remedy analgesia within 48 h postoperatively.
- The incidence of adverse events such as nausea and vomiting, agitation, and respiratory depression within 48 h after surgery.

### Statistical analysis

The primary observation indicators in the present study were the VAS at rest and with a cough at 1, 6, 12, 24, and 48 h postoperatively. The sample size was calculated based on the results of a pilot study with five patients in each group. In the pilot study, the VAS (mean ± standard deviation [SD]) at rest 1 h postoperatively in groups C, T, and E were 1.98 ± 0.58, 1.34 ± 0.62, and 1.40 ± 0.61, respectively, with the test levels set at *α* = 0.05 and 1 − *β* = 0.80, and the ratio of the number of cases in the three groups was 1:1:1. Using the SAS 9.4 statistical software (SAS Institute, Cary, NC, USA), the sample size of 20 patients in each of the three groups was calculated, and then, based on the 20% dropout rate, it was finally calculated that each group should have at least 24 patients.

The statistical analysis was performed using the statistical software IBM SPSS Statistics for Windows, Version 22.0 (IBM Corp., Armonk, NY, USA). The measurement data were expressed as mean ± standard deviation ($\overline{x}$ ± ***s***). One-way ANOVA was used to compare the satisfactory measurement data to the normal distribution among the three groups. The Kruskal–Wallis *H* test was used for the unsatisfactory data with normal distribution or variance disparity. The Friedman test was used for comparison among multiple time points within groups not satisfied with the normal distribution. The countable data were expressed as the frequency or rate, and the Pearson’s chi-squared test was used for comparison among the three groups. *p* < 0.05 was considered statistically significant.

## Results

### General characteristics

**Fig. 2:**
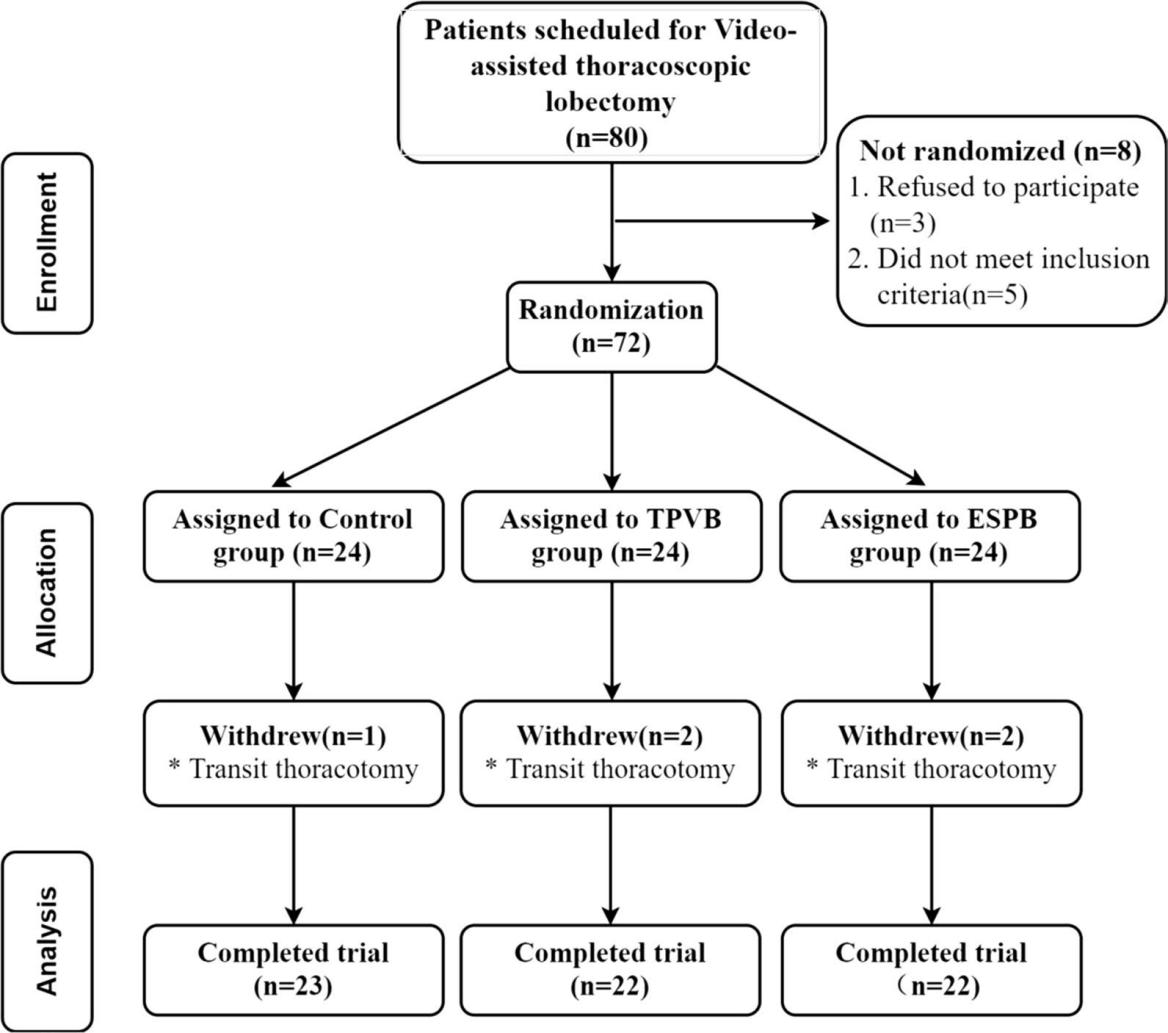
The flow chart of the participants. *Note* TPVB: Thoracic paravertebral nerve block; ESPB: Erector spinae plane block

Eighty patients undergoing elective thoracoscopic pulmonary lobectomy under general anesthesia with tracheal intubation were selected for the present study. Three patients were excluded because they refused to participate in the study, and five patients were excluded because they did not meet the inclusion criteria for the study (*n* = 2, age > 70 years; *n* = 3, BMI > 30 kg/m^2^). Seventy-two patients were finally enrolled in the present study. Five patients were excluded from the final data analysis during the study due to intraoperative conversion to open-thoracic surgery (Fig. 2). There was no statistically significant difference in gender, age, BMI, ASA classification, operation duration, and anesthesia duration among the three groups of patients (*p* > 0.05) (Table 1).

**Table 1:** Comparison of general data of patients in the three groups

	Group C (*n* = 23)	Group T (*n* = 22)	Group E (*n* = 22)	*F/χ*^*2*^	*p*
Gender (Male/Female)	11/12	10/12	11/11	0.091	0.955
Age (yr)	52.13 ± 6.55	54.32 ± 6.56	54.41 ± 7.61	0.787	0.459
Body mass index (kg/m^2^)	25.01 ± 2.45	25.47 ± 2.65	25.56 ± 3.01	0.267	0.767
ASA(I/II)	7/16	9/13	7/15	0.639	0.727
Time of operation (min)	124.87 ± 10.34	126.05 ± 6.81	126.82 ± 7.56	0.307	0.737
Anesthesia time (min)	161.57 ± 9.42	163.86 ± 8.03	163.23 ± 7.38	0.458	0.635

The general data of the three groups of patients were expressed as mean ± standard deviation or absolute value. Group C was the control group; Group T was the thoracic paravertebral nerve block group; Group E was the erector spinae plane block group; ASA: American Society of Anesthesiologists. There was no statistically significant difference in the general data among the three groups

### Intraoperative sufentanil consumption, anesthesia awakening time, and extubation time

#### Intraoperative sufentanil consumption

Intraoperative sufentanil consumption was significantly lower in patients in groups T and E compared with group C (32.77 ± 5.57 μg, 33.86 ± 2.88 μg vs. 41.09 ± 3.46 μg; *F* = 27.140, *p* < 0.001). There was no significant difference in intraoperative sufentanil consumption between groups T and E (*p* > 0.05) (Table 2).

**Table 2:** Comparison of intraoperative sufentanil consumption, anesthesia recovery time and extubation time among the three groups ($\overline{x}$ ± *s*)

	Group C (*n* = 23)	Group T (*n* = 22)	Group E (*n* = 22)	*F*	*p*
Sufentanil consumption (μg)	41.09 ± 3.46	32.77 ± 5.57*	33.86 ± 2.88*	27.140	< 0.001
Anesthesia recovery time (min)	23.83 ± 4.17	19.09 ± 3.46*	19.50 ± 4.30*	9.760	< 0.001
Anesthesia extubation time (min)	29.22 ± 4.98	23.64 ± 4.22*	24.64 ± 4.28*	9.839	< 0.001

Group C was the control group, group T was the paravertebral nerve block group, and group E was the erector spinal plane block group. * is compared with group C, *p* < 0.05

#### Anesthesia awakening time

Compared with group C, the anesthesia awakening time was significantly shorter in groups T and E (19.09 ± 3.46 min, 19.50 ± 4.30 min vs. 23.83 ± 4.17 min; *F* = 9.760, *p* < 0.001), and there was no statistically significant difference in the anesthesia awakening time between groups T and E (*p* > 0.05) (Table 2).

#### Extubation time

The extubation time was significantly shorter in groups T and E compared with group C (23.64 ± 4.22 min, 24.64 ± 4.28 min vs. 29.22 ± 4.98 min; *F* = 9.839, *p* < 0.001), but the difference was not statistically significant when compared between groups T and E (*p* > 0.05) (Table 2).

### VAS at different time points and different statuses after surgery

#### At rest

The comparison between groups showed that the VAS of patients in group T were lower than those in group C at 1, 6, and 12 h after surgery, and the differences were statistically significant (*p* < 0.05). The VAS of patients in group E were lower than those in group C at 1 and 6 h after surgery, and the differences were statistically significant (*p* < 0.05). There was no significant difference in VAS among the three groups at 24 and 48 h after surgery (*p* > 0.05). The intra-group comparison showed that the VAS in the three groups were statistically significant at different time points after surgery (group C: χ2 = 81.408, *p* < 0.001; group T: χ2 = 83.434, *p* < 0.001; group E: χ2 = 84.853, *p* < 0.001), the pair-wise comparison showed that VAS gradually increased with time, and the differences between each time point were statistically significant (*p* < 0.05) (Table 3).

**Table 3:** Comparison of VAS scores at different time points and in different states after surgery between the two groups

Status	Group	Number of cases	VAS_1h_	VAS_6h_	VAS_12h_	VAS_24h_	VAS_48h_
Tranquillization	Group C	23	1.91 ± 0.67	2.70 ± 0.56^a^	3.57 ± 0.73^ab^	4.09 ± 0.73^abc^	4.96 ± 0.88^abcd^
	Group T	22	1.27 ± 0.70^*^	1.91 ± 0.61^a*^	2.64 ± 0.73^ab*^	3.91 ± 0.75^abc^	4.86 ± 0.64^abcd^
	Group E	22	1.32 ± 0.65^*^	2.05 ± 0.58^a*^	3.05 ± 0.58^ab^	4.05 ± 0.65^abc^	4.91 ± 0.68^abcd^
	H		9.942	18.131	15.997	0.655	0.281
	*P*		0.007	< 0.001	< 0.001	0.721	0.869
Cough	Group C	23	3.17 ± 0.72	3.61 ± 0.58^a^	4.35 ± 0.71^ab^	4.91 ± 0.60^abc^	5.70 ± 0.76^abcd^
	Group T	22	2.32 ± 0.89^*^	2.77 ± 0.69^a*^	3.27 ± 0.63^ab*^	4.86 ± 0.64^abc^	5.55 ± 0.60^abcd^
	Group E	22	2.45 ± 0.74^*^	2.86 ± 0.71^a*^	3.73 ± 0.63^ab*^	4.91 ± 0.61^abc^	5.64 ± 0.49^abcd^
	H		12.671	16.575	20.364	0.102	1.290
	*P*		0.002	< 0.001	< 0.001	0.950	0.525

Group C was the control group, group T was the paravertebral nerve block group, group E was the erector spinal muscle plane block group, VAS: visual analogue score; ^a^ is compared with 1 h *p* < 0.05, ^b^ is compared with 6 h *p* < 0.05, ^c^ is compared with 12 h *p* < 0.05, ^d^ is compared with 24 h *p* < 0.05, * is compared with C group *p* < 0.05

#### With cough

Comparison between groups showed that the VAS in patients in groups T and E were lower than those in group C at 1, 6, and 12 h postoperatively, and the differences were statistically significant (*p* < 0.05), while the differences in the VAS of patients among the three groups at 24 and 48 h postoperatively were not statistically significant (*p* > 0.05). Within-group comparisons showed that the differences in VAS at different time points after surgery were statistically significant in all three groups (Group C: *χ*2 = 78.473, *p* < 0.001; Group T: *χ*2 = 75.871, *p* < 0.001; Group E: *χ*2 = 77.674, *p* < 0.001), and a two-by-two comparison showed that VAS gradually increased with time, and the differences between all time points were statistically significant (*p* < 0.05) (Table 3).

#### Postoperative sufentanil consumption and flurbiprofen ester remedy dose

Compared with group C, sufentanil consumption in the analgesic pump 48 h after operation in groups T and E were significantly reduced (57.05 ± 2.21 μg, 60.09 ± 3.05 μg vs. 64.09 ± 7.07 μg; H = 18.654, *p* < 0.001), the postoperative sufentanil consumption in group E was more than that in group T (*p* < 0.05) (Table 4). There was no statistically significant difference in the dose of flurbiprofen ester for remedy analgesia among the three groups (H = 1.376, *p* = 0.503) (Table 4).

**Table 4:** Comparison of postoperative sufentanil consumption and flurbiprofen ester recovery dose in the three groups (± s)

	Group C (*n* = 23)	Group T (*n* = 22)	Group E (*n* = 22)	*H*	*p*
Sufentanil consumption (μg)	64.09 ± 7.07	57.05 ± 2.21^*^	60.09 ± 3.05^*#^	18.654	< 0.001
Remediation dose of flurbiprofen axate (mg)	106.52 ± 52.88	86.36 ± 35.13	95.45 ± 43.40	1.376	0.503

Group C was the control group, group T was the paravertebral nerve block group, and group E was the erector spinal plane block group. * is compared with group C, *p* < 0.05, # is compared with group T, *p* < 0.05

### The incidence of postoperative adverse events

There were no adverse events such as pneumothorax, nerve injury, or local hematoma in the three groups. There was no statistically significant difference in the incidence of nausea, vomiting, and agitation within 48 h after operation among the three groups of patients (*p* > 0.05) (Table 5). There was no postoperative respiratory inhibition in the three groups.

**Table 5:** Comparison of postoperative adverse reactions among the three groups [Number (%)]

	Group C (*n* = 23)	Group T (*n* = 22)	Group E (*n* = 22)	χ^2^	*p*
Nausea and vomiting	6 (26.09)	4 (18.18)	3 (13.64)	1.146	0.564
Dysphoria	5 (21.74)	3 (13.64)	2 (9.09)	1.460	0.482

Group C was the control group, group T was the paravertebral nerve block group, and group E was the erector spinal plane block group

## Discussion

The present study showed that a single preoperative ultrasound-guided TPVB resulted in lower intraoperative sufentanil consumption and shorter awakening time and extubation time than the control group without nerve block, suggesting that general anesthesia combined with a single TPVB during thoracoscopic pulmonary lobectomy could provide effective intraoperative analgesia and good anesthetic awakening. The possible reasons were speculated as, TPVB is a nerve block technique in which local anesthetic is injected directly into the thoracic paravertebral space to block the thoracic spinal nerve and the branches as well as the sympathetic trunk, and the local anesthetic could spread cranially and caudally through the loose connective tissue of the thoracic paravertebral space [15], as well as laterally to the intercostal and epidural spaces [16], so it could provide analgesia comparable to that of the thoracic segmental epidural block.

The results of the present study revealed that a single preoperative ultrasound-guided ESPB also reduced the intraoperative sufentanil consumption and shortened the awakening and extubation time compared with the control group without nerve block, suggesting that general anesthesia combined with a single ESPB could provide effective intraoperative analgesia and good awakening in patients undergoing thoracoscopic pulmonary lobectomy. It was assumed that the possible reasons were, after the preoperative administration of a single ESPB, local anesthetics injected into the interstices of the erector spinae and transverse spinal processes could spread cranially and caudally along with the deep layers of the thoracolumbar fascia, blocking the ventral and dorsal branches of the thoracic spinal nerves and the traffic branches of the adjacent segments, and some drugs could also block the sympathetic nerves and result in suppression of visceral pain [17].

In the present study, the VAS at rest and with a cough at 1, 6, and 12 h postoperatively were lower in group T than in group C at the same time point. The VAS at rest at 1 and 6 h postoperatively and with a cough at 1, 6, and 12 h postoperatively were lower in group E than in group C at the same time point. The consumption of sufentanil in the analgesic pump within 48 h after operation was significantly lower in groups T and E than in group C. These indicated that the ultrasound-guided preoperative single TPVB or ESPB for thoracoscopic pulmonary lobectomy could provide effective postoperative analgesia, reduce the VAS of the postoperative pain, and reduce the dose of postoperative sufentanil and remedial analgesics. Previous studies have shown that the duration of analgesia is 12–24 h for TPVB [18] and 10–12 h for ESPB [19]. It was found in the present study that the differences in VAS at rest and with a cough at 24 and 48 h after surgery were not statistically significant among the three groups, suggesting that a single administration of 30 ml of 0.5% ropivacaine neither in TPVB nor in ESPB could meet the requirement for analgesia throughout the postoperative period in patients undergoing thoracoscopic surgery. Whether the addition of adjuvants such as dexmedetomidine or dexamethasone to local anesthetics can prolong the duration of action of TPVB or ESPB will be investigated in further studies.

TPVB injects the local anesthetic into the paravertebral space, which is anatomically close to the pleura. There is a risk of accidentally injecting the local anesthetic into the blood vessels or hemopneumothorax with traditional blind penetration. The ESPB technique is to inject the local anesthetic between the plane of the erector spinae and the transverse process of the thoracic spine, which can greatly avoid the occurrence of pneumothorax due to the anatomical positioning of the transverse process of the thoracic spine with improved safety [20].

In the present study, TPVB or ESPB was performed under ultrasound guidance, which could clearly confirm the location of the pleural, transverse, and paravertebral spaces, observe the direction of the puncture needle and monitor the diffusion of the local anesthetic in real-time, which significantly improved the success rate of the block [21] and reduced the occurrence of adverse reactions such as pneumothorax and hemothorax [22]. In the present study, no adverse events such as pneumothorax, nerve injury, local hematoma, or block failure occurred in patients in groups T and E. This indicated that the perioperative application of TPVB and ESPB in patients undergoing thoracoscopic surgery was safe and effective.

Santonastaso et al. [23] found that in patients undergoing perioperative analgesia for thoracic surgery, the ultrasound-guided TPVB at the T4 and T5 levels resulted in the diffusion of local anesthetic up to the T2 level in the head and down to the T7 level in the caudal end. Despite the exact site of action of TPVB, the blocking range of one interspace is limited, and Vogt et al. [24] concluded that multiple injections would unnecessarily expose the patients to additional risks associated with puncture. Comparatively, ESPB has a wide range of action, and Ueshima et al. [25] reported that local anesthetic injected into the deep T5 transverse process of the erector spinae could spread over five intervertebral spaces. Chin et al. [20] found that injection of 20 ml of 0.5% ropivacaine into the plane of the erector spinae could spread at least three vertebral levels from the injection site to the cephalic end and four vertebral levels to the caudal end. In the present study, because the nerve block was performed after the induction of anesthesia in patients, the anesthetic planes of TPVB and ESPB were not detected. The possibility of nerve block failure could not be completely excluded, which was a shortcoming of the present study.

The limitations in the present study and related follow-up studies were: (1) the present study was an exploratory single-center study with a small sample size, and the generalizability of the findings was uncertain. Therefore, follow-up studies should be conducted to verify the present study’s findings by expanding the sample size and conducting multi-center studies. (2) It was found in the present study that a single administration of 30 ml of 0.5% ropivacaine in TPVB or ESPB was not enough to meet the requirement of full postoperative analgesia in patients undergoing thoracoscopic surgery. Thus, the addition of adjuvants such as dexmedetomidine or dexamethasone to local anesthetics to prolong the duration of action of TPVB or ESPB should be investigated subsequently.

## Conclusion

In conclusion, preoperative ultrasound-guided single TPVB and ESPB for thoracoscopic pulmonary lobectomy could reduce the VAS of postoperative pain in patients and decrease the perioperative sufentanil and postoperative remedial analgesic medications.

## Data Availability

The datasets used and/or analyzed during the current study available from the corresponding author on reasonable request.

## Abbreviations

ASA: American Society of Anesthesiologists
BIS: Bispectral index
BMI: Body mass index
BR: Breathing rate
ECG: Electrocardiogram
ESPB: Erector spinae plane block
HR: Heart rate
MAP: Mean arterial pressure
NIBP: Non-invasive blood pressure
PetCO2: End-tidal carbon dioxide partial pressure
SBP: Systolic blood pressure
SD: Standard deviation
SpO2: Pulse oxygen saturation
TGF-β: Transforming growth factor-β
TPVB: Thoracic paravertebral nerve block
VAS: Visual analogue score
VEGF: Vascular endothelial growth factor
